# Lymphoblastic Involvement of the Bone Marrow as a Cause of Superscan Appearance in ^18^F-Fluorodeoxyglucose Positron Emission Tomography/ Computed Tomography

**DOI:** 10.4274/mirt.galenos.2019.42104

**Published:** 2020-04-29

**Authors:** Zehra Pınar Koç, Pelin Özcan Kara, Aydan Akdeniz, Mehmet Yaldız

**Affiliations:** 1 Mersin University Faculty of Medicine, Department of Nuclear Medicine, Mersin, Turkey; 2 Mersin University Faculty of Medicine, Department of Hematology, Mersin, Turkey; 3 Mersin University Faculty of Medicine, Department of Pathology, Mersin, Turkey

**Keywords:** Superscan, FDG, PET/CT, lymphoma

## Abstract

The ^18^F-fluorodeoxylucose (FDG) positron emission tomography (PET) is the gold standard imaging modality in the staging of lymphoma. The superscan appearance in the FDG PET/computerized tomography (CT) imaging might be because of benign diseases or malignant infiltrations. This case report presents lymphomatous blastic infiltration as a cause of superscan appearance in ^18^F-FDG PET/CT.

## Abstract (tr)

^18^F-florodeoksiglikoz (FDG) pozitron emisyon tomografisi/bilgisayarlı tomografi (PET/BT) lenfoma evrelemesinde altın standard görüntüleme yöntemidir. FDG PET/BT’de supersken görünümü benign hastalıklardan dolayı veya dokuların malign hastalıklarla tutulmasından kaynaklanabilir. Bu olgu sunumunda FDG PET/BT’de lenfomatöz blastik infiltrasyon sonucu olan supersken görünümü sunulmaktadır

## Figures and Tables

**Figure 1:**
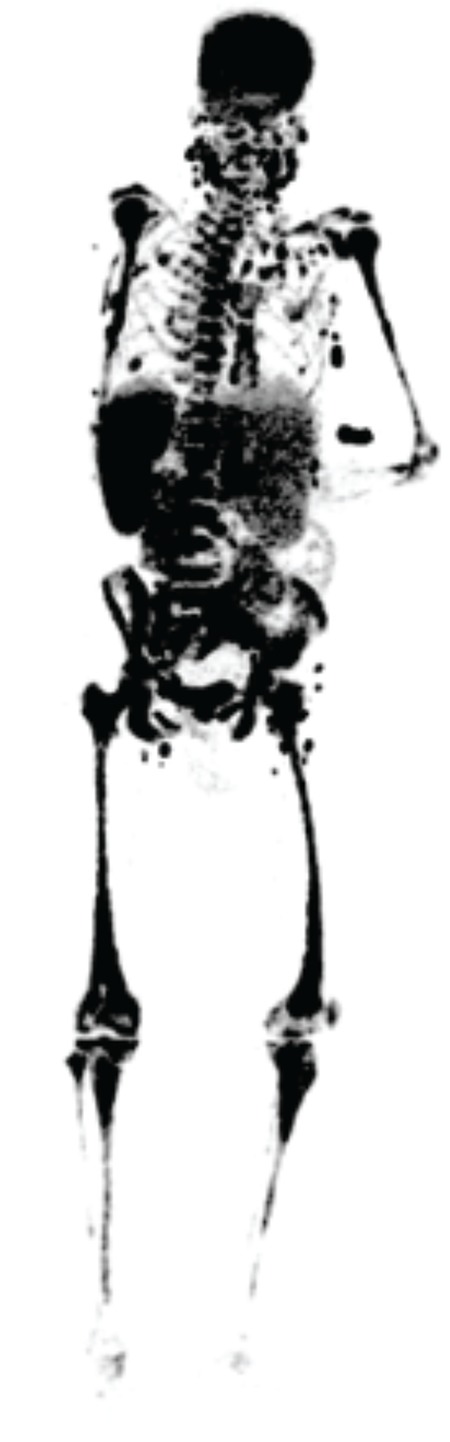
Multiple intensity projection image of the fluorodeoxylucose (FDG) positron emission tomograph/computerized tomography (PET/CT) demonstrating significant uptake in bone marrow, spleen and lymph nodes and faint physiological activity in brain and liver without other soft tissue. Fifty-four-year-old female patient presented with anemia, leukopenia and trombositopenia. The patient was referred for ^18^F-FDG PET/CT imaging and bone marrow biopsy procedure. The PET/CT imaging was performed after a fasting period of 12 hours and the blood glucose level was 111 mg/dL. The imaging was performed 60 minutes after intravenous administration of 7.7 mCi ^18^F-FDG in craniocaudal direction in 3D acquisition mode for 1 min per bed position with nondiagnostic CT scan for attenuation correction with oral contrast administration. The imaging revealed multiple servical, mediastinal, abdominal lymph nodes and severe bone marrow activity accumulations suggesting the infiltration of the disease as well as diminished activity in the soft tissues, physiological uptake in the brain, liver and spleen (Figure 1).

**Figure 2:**
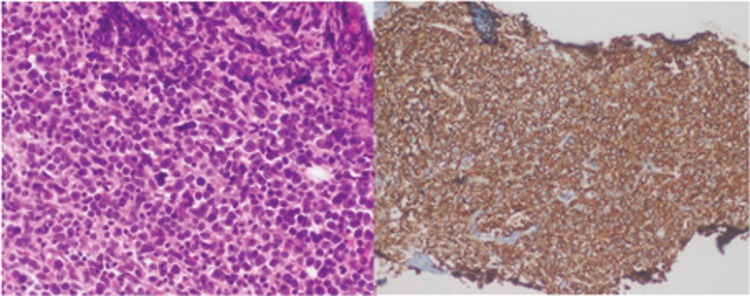
Pathology images of bone marrow biopsy show bone marrow infiltration of lymphoblastic cells with hematoxylene and eosine (left) and CD20 staining (right). The bone marrow biopsy revealed complete blastic (100%) infiltration of the bone marrow (Figure 2) and diagnosis of B cell lymphoma. The superscan imaging examples in the literature were usually due to the malignant tumor infiltration of the bone marrow or hepatic infiltration. According to a review analysis, the possible causes of superscan appearance in the FDG PET/CT imaging of the bone marrow might be due to the benign and malignant pathologies including the colony stimulating factors, pyrexia due to infection, primary or secondary hyperparathyroidism (1). Additionally, diffuse hepatic activity as a consequence of hepatic superscan in the FDG PET/CT was reported in the hepatic lymphoma (2) and hepatic angiosarcoma (3), previously. The superscan appearance obscures some findings and this causes false negative misinterpretation of some of the malignant lesions as well. There were two previously reported cases with diffuse large B cell lymphoma infiltration of the liver (4,5). These cases had significantly decreased physiologic uptake in the brain, cardiac and renal tissues as well. Parida et al. (6) reported a case with superscan acute lymphoblastic lymphoma with slight physiological uptakes in liver and spleen. This present case showed significant increased activity in bone marrow and spleen and superscan appearance as a result of malignant infiltration of “acute lymphoblastic lymphoma” of the tissues which was documented by bone marrow biopsy results.
